# Cytotoxic Potential of Bio-Silica Conjugate with Different Sizes of Silver Nanoparticles for Cancer Cell Death

**DOI:** 10.3390/ma15124074

**Published:** 2022-06-08

**Authors:** Mohamed S. Hamdy, Serag Eldin I. Elbehairi, Ali A. Shati, Hisham S. M. Abd-Rabboh, Mohammad Y. Alfaifi, Khaled F. Fawy, Hala A. Ibrahium, Saad Alamri, Nasser S. Awwad

**Affiliations:** 1 Department of Chemistry, Faculty of Science, King Khalid University, P.O. Box 9004, Abha 61413, Saudi Arabia; mhsaad@kku.edu.sa (M.S.H.); habdrabboh@kku.edu.sa (H.S.M.A.-R.); khossayn@kku.edu.sa (K.F.F.); 2 Department of Biology, Faculty of Science, King Khalid University, P.O. Box 9004, Abha 61413, Saudi Arabia; serag@kku.edu.sa (S.E.I.E.); aaalshati@kku.edu.sa (A.A.S.); alfaifi@kku.edu.sa (M.Y.A.); habrahem@kku.edu.sa (H.A.I.); saralomari@kku.edu.sa (S.A.)

**Keywords:** silver nanoparticles, bio-based silica, cytotoxicity, apoptosis, necrosis, anti-cancer

## Abstract

Well-defined silver nanoparticles were doped into bio-based amorphous silica (Ag-b-SiO_2_) with different silver contents (from 2 to 20 wt%) by a solvent-free procedure. The four as-synthetized samples were hydrogenated at 300 °C to ensure the formation of zero-valent Ag nanoparticles. The prepared samples were characterized by X-ray powder diffraction (XRD), elemental analysis, N_2_ sorption measurements, scanning electron microscopy (SEM), X-ray photoelectron spectroscopy (XPS), and high-resolution transmission electron microscopy (HR-TEM). The characterization data confirmed the formation of well-defined zero-valent silver nanoparticles in the range of 3–10 nm in the low-loading samples, while in high-loading samples, bulky particles of silver in the range of 200–500 nm were formed. The in vitro cytotoxic activities of the Ag-b-SiO_2_ samples were tested against the tumor cell lines of breast (MCF-7), liver (HepG2), and colon (HCT 116) over a concentration range of 0.01 to 1000 g. The prepared samples exhibited a wide range of cytotoxic activities against cancer cells. An inverse relationship was observed between the silver nanoparticles’ size and the cytotoxic activity, while a direct relationship between the silver nanoparticles’ size and the apoptotic cell death was noticed.

## 1. Introduction

Despite the significant efforts of scientists to control cancer, there is a global increase in cancer patients around the world [1]. Moreover, the World Health Organization (WHO) indicated cancer as a main reason for death around the world, almost 10 M deaths in 2020 caused by different types of cancer [2]. Therefore, the creation and/or development of unusual treatments for cancer is crucial. The pioneering work of El-Sayed [3,4] indicated the high potential of metallic nanoparticles, e.g., gold and silver, in fighting cancer cells. Many articles have reported the toxic behavior of Au [5,6] and Ag nanoparticles [7,8] against cells of various cancer types. Silver nanoparticles were applied either as solo metallic particles or supported zero-valent nanoparticles. Several methods have been reported to prepare the solo zero-valent nanoparticles such as chemical [9], physical [10], and even biological [11] synthesis.

However, there is a concern about the stability of the presence of silver nanoparticles in a solution for a relatively long time. Silver nanoparticles can be agglomerated [12] and hence lose their activity as a toxic agent. The stability of the nanoparticles can be improved when located on a suitable support such as silica [13], alumina [14], zeolite [15], and, recently, on graphene oxide [16].

**Figure 1:**
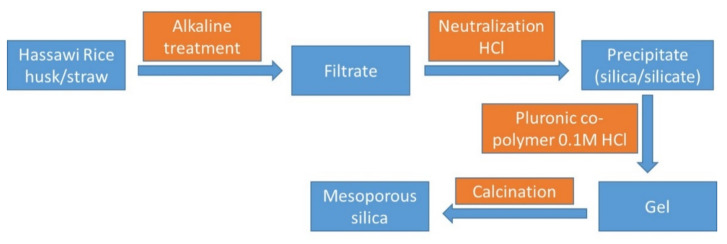
The valorization of Hassawi rice husk/straw into different products [17]. In the current work, the research team utilizes the bio-based silica to prepare anti-cancer compounds.

Recently, the current research team reported the extraction and purification of amorphous silica from Hassawi rice husk/straw [17] through few simple treatment steps (Figure 1); silicate ions could be formed, which could be utilized to produce amorphous spheres of mesoporous silica [17]. The extracted bio-based silica was used to support silver nanoparticles in one-step synthesis by using a solution of silver nitrate without the use of any organic solvents. The motivation behind the current study is to introduce a cost-effective material that can be applied as anti-cancer materials. The synthesis and the characterization of the silver nanoparticles supported on amorphous bio-based silica are reported; moreover, the anti-cancer activity [18,19,20,21,22,23] of the prepared material against several cancer cells is discussed.

## 2. Results

### 2.1. The Characterization of Ag-Doped Amorphous Silica

**Figure 2:**
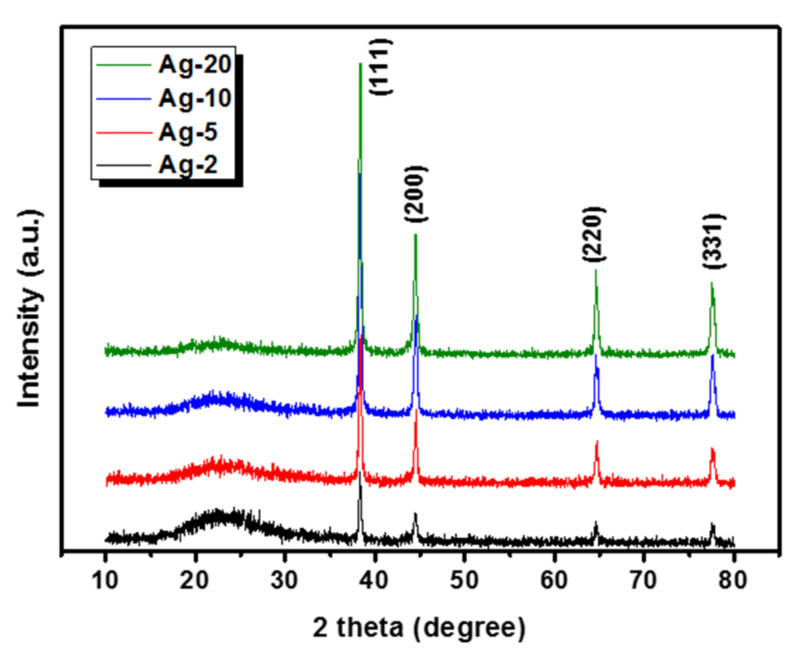
XRD patterns of the prepared silver nanoparticles supported on bio-based silica.

XRD analysis was applied to study the crystallinity of the prepared samples, and the obtained patterns are plotted in Figure 2. In the entire samples, a very broad band was observed near 23° 2θ as an indication for the amorphous nature of the silica support [23]. Moreover, four beaks were located at the locations of 38.4°, 44.7°, 64.5°, and 78.0° 2θ, which correspond to the FCC (the face-centered cubic) planes of (111), (200), (220), and (331), which can be attributed to the presence of zero-valent silver nanoparticles according to the JCPDS database card no# 04-0783 [24]. Moreover, the peak intensity of silver nanoparticles increased with the loading, which is consistent with increasing silver content in the samples.

ICP analysis was applied to investigate the exact amount of silver in each sample; on the other hand, N_2_ sorption analysis was used to study the texture properties of the prepared samples. The obtained results are listed in Table 1.

The elemental analysis of the prepared samples showed that almost 70–78% of the silver amount added to the synthesis mixture were obtained in the final solid sample. Moreover, the texture properties showed that the surface area of the prepared samples was found to be increased from 580 m^2^/g in the bare bio-based silica sample to 620–637 m^2^/g as an indication for the effect of silver nanoparticles on the integrity of the prepared samples. Moreover, the pore volume and pore diameter were found to be decreased due to the presence of silver nanoparticles in the pores of the mesoporous bio-based silica.

**Figure 3:**
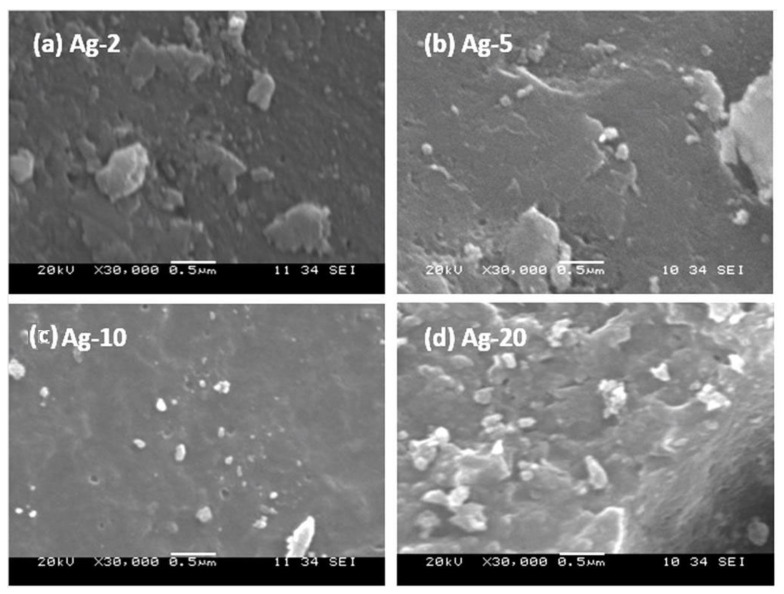
SEM micrographs of the prepared silver nanoparticles supported on amorphous bio-based silica samples.

The morphology of the prepared samples’ surface was observed by the SEM technique, and the micrographs of the prepared samples are presented in Figure 3. The micrographs of the small loading samples (i.e., Ag-2 and -5) showed only the clean surface of the bio-based amorphous silica. More importantly, no other phase(s) was/were detected. However, the micrographs of the high-loading samples (Figure 3, Ag-10 and -20) showed some extra framework silver nanoparticles.

**Figure 4:**
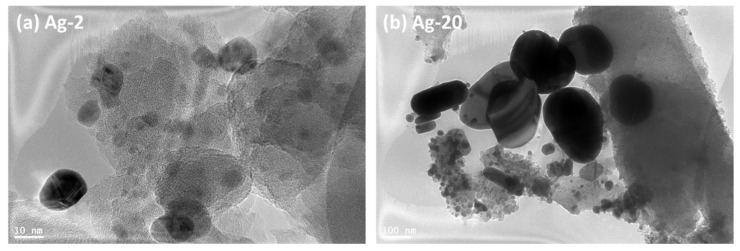
The HR-TEM micrographs of Ag-2 and Ag-20 samples.

The size and distribution of silver nanoparticles were investigated through the HR-TEM technique, and the obtained micrographs are presented in Figure 4. The micrographs clearly showed the difference in the location and the size of silver nanoparticles. In the Ag-2 sample, the majority of silver nanoparticles were embedded and well-distributed in the silica support; moreover, the size of the nanoparticles ranged between 3 and 10 nm with a good distribution within the silica support. However, in the Ag-20 sample, the micrograph showed a considerable part of silver nanoparticles located inside the silica support, while some other agglomerated silver particles can also be observed. The size distribution in Ag-20 seemed to be very broad, the average of the embedded nanoparticles seemed to be 3–10, while the size of the extra framework particles ranged from 200 to 500 nm, which agreed with the results of the XRD and SEM study.

**Figure 5:**
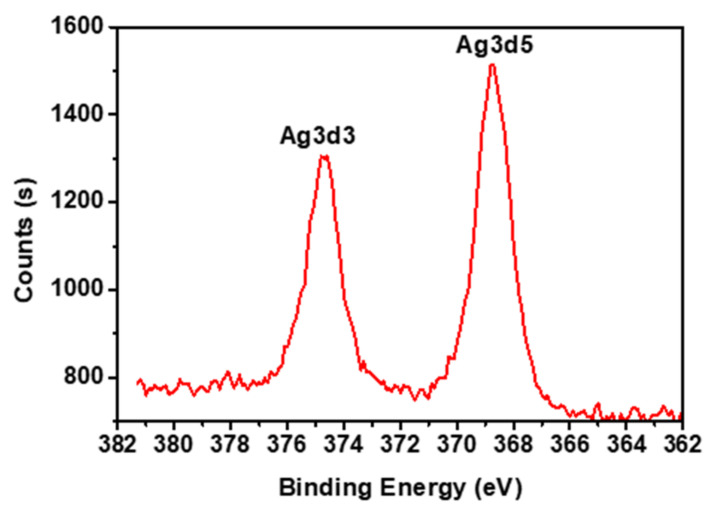
XPS analysis of Ag-2 sample.

Finally, the oxidation state of the silver nanoparticles was investigated through XPS analysis (Figure 5). XPS analysis showed two peaks at 368.1 eV and 370 eV, which corresponded to Ag 3d_3/2_ and Ag 3d_5/2_, respectively, as an indication for the presence of silver nanoparticles in the zero-state oxidation state [25,26].

### 2.2. The Anti-Cancer Activity

The SRB assay was used to assess the in vitro cytotoxic activities of the prepared Ag-b-SiO_2_ samples against the tumor cell lines MCF-7, HepG2, and HCT 116 over a concentration range of 0.01 to 1000 g. The obtained results showed negligible cytotoxic activity for the bare bio-based silica sample against the three investigated cancer cells.

**Figure 6:**
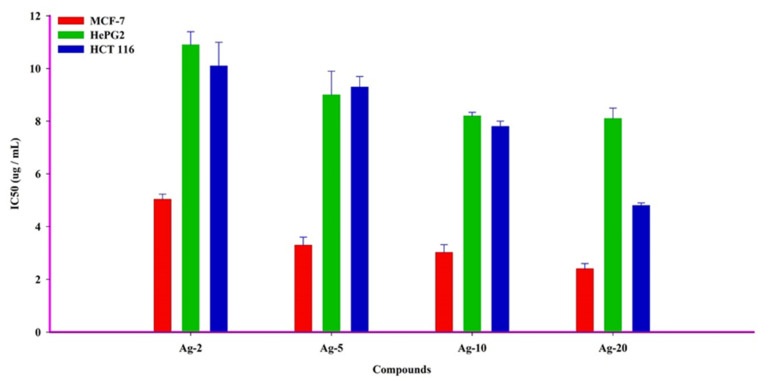
IC_50_ values of the prepared samples after treating different human cancer cell lines (MCF-7, HePG2, and HCT 116).

**Figure 7:**
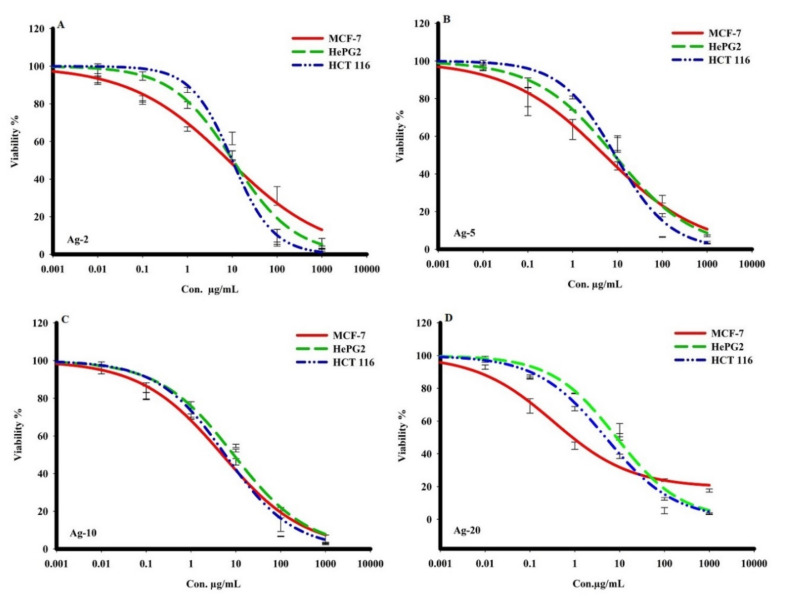
The cytotoxic activity of the prepared Ag-b-SiO_2_ in MCF-7, HepG2, and HCT 116 human cancer cells. For 72 h, cells were exposed to various doses of isolated materials. SRB staining was used to assess cell viability.

On the other hand, the obtained results showed that the prepared Ag-b-SiO_2_ samples exhibited a wide range of cytotoxic activities against cancer cells. Ag-20 showed the most potent cytotoxic traits in human breast adenocarcinoma metastatic cells (MCF-7), with an IC_50_ of 2.4 ± 0.2 g/mL, and other compounds Ag-2, Ag-5, and Ag-10 exhibited the most significant killing effect against the same cancer cells (MCF-7) with IC_50_s of 5.03 ± 0.2, 3.3 ± 0.3, and 3.02 ± 0.3 g/mL, respectively. In comparison to other cancer cells, the compound Ag-20 showed strong cell killing with an IC_50_ of 4.8 ± 0.1 g/mL on human colon adenocarcinoma cells (HCT 116). Furthermore, Ag-2, Ag-5, and Ag-10 samples showed cytotoxicity against HepG2 and HCT 116 cancer cells, with IC_50_s ranging from 7.8 ± 0.2 to 10.9 ± 0.5 g/mL (Table 2, and Figure 6 and Figure 7). The obtained results clearly showed that there was a great effect of the nanoparticle size on the cytotoxic activity of the prepared samples.

**Figure 8:**
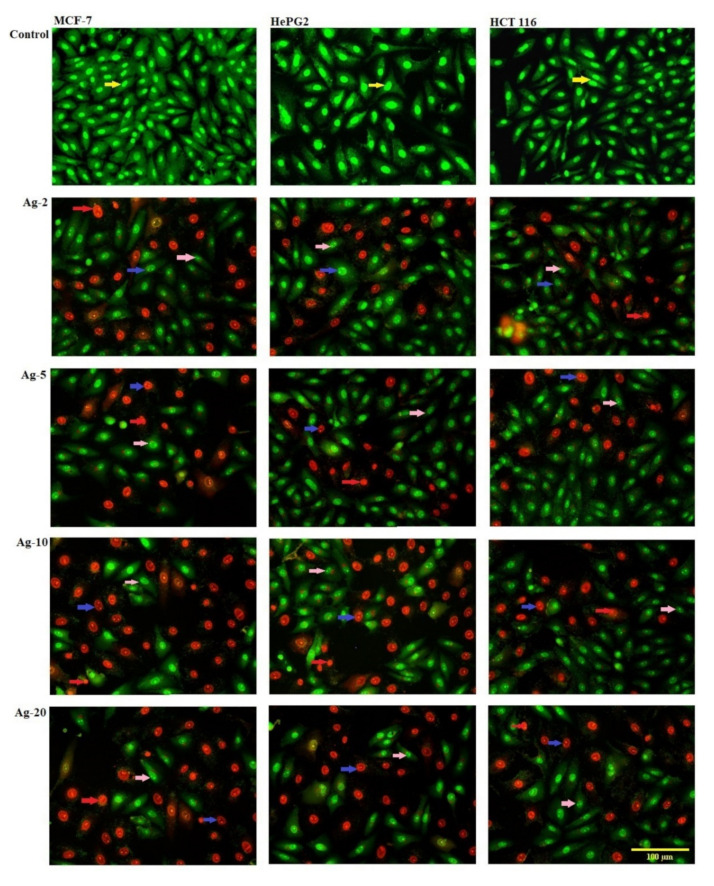
The effect of nanosilver compounds treatment on the apoptosis and necrosis of MCF-7, HepG2, and HCT 116 human tumor cells was evaluated using acridine orange (AO) and ethidium bromide (EB) staining after 48 h of treatment, inducing various nuclear changes such as chromatin fragmentation and condensation at 200×. Yellow arrows indicate live cells, pink arrows indicate early apoptotic cells, red arrows indicate necrotic cells, and blue arrows indicate late apoptotic cells.

**Figure 9:**
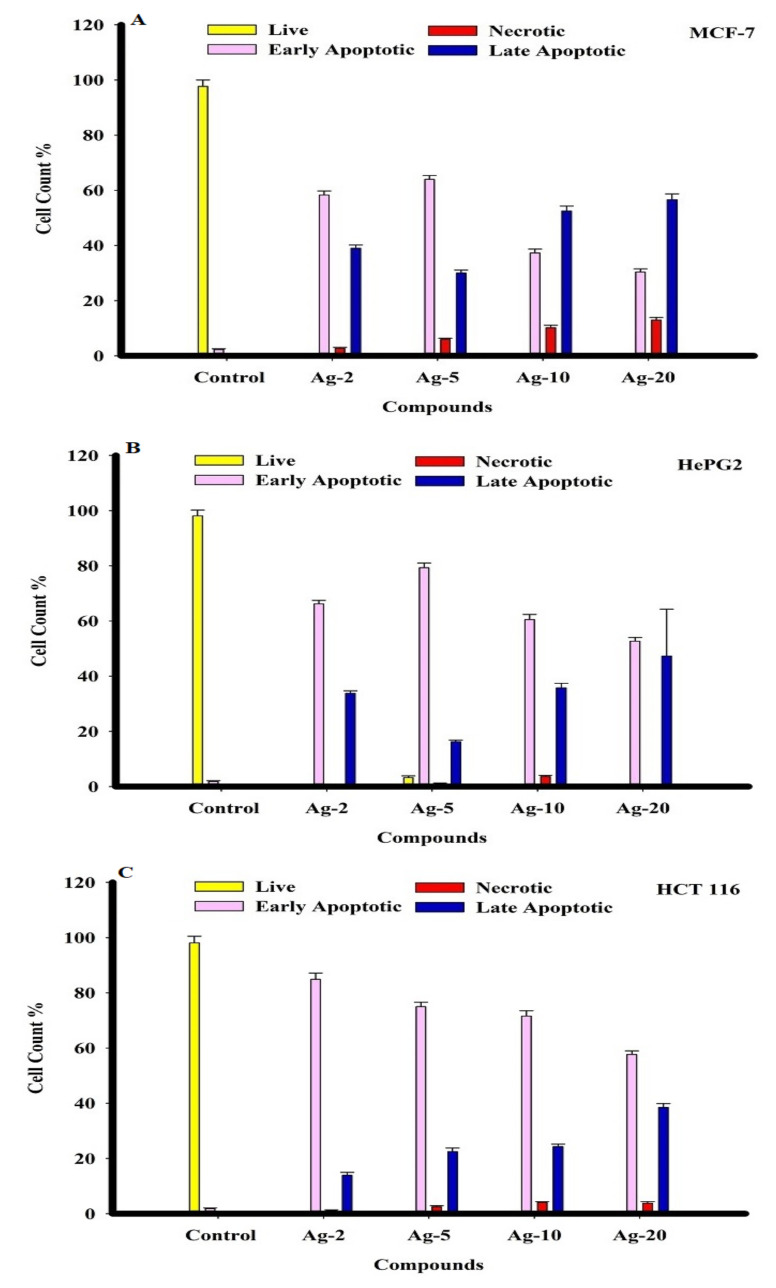
The percentage of apoptotic/necrotic cells of MCF-7, HePG2, and HCT 116 tumor cells was determined 48 h after treatment with silver nanoparticles.

The cytotoxicity of the prepared Ag-b-SiO_2_ samples in MCF-7, HepG2, and HCT116 human cancer cells was monitored in a time frame of 72 h, and cells were exposed to various doses of the prepared samples. Dual fluorescent staining of acridine orange/ethidium bromide (AO/EtBr) was applied to recognize MCF-7, HepG2, and HCT 116 cancer cells. The cancer cells were treated for 48 h before being investigated under a fluorescent microscope. The viability of the cell control was indicated by regular green staining with normal, round, intact nuclei and cytoplasm. After treating all cancer cells with Ag-5, a higher rate of cell death was observed in the early apoptosis pathway, even though the percentage was higher than Ag-5 after treating HCT116 cancer cells with Ag-2. In addition, Ag-10 caused a high percentage of MCF-7, HePG2, and HCT 116 cells to necrotize. After treatment with Ag-20, all cancer cells showed the highest rate of late apoptosis (Figure 8 and Figure 9).

## 3. Discussion

Generally, the obtained results showed the inhibition of the three different kinds of cancer cells through apoptotic death for the cells. The mechanism of inhibition was reported earlier. In the case of colon cancer cells (HCT 116), it was reported that silver nanoparticles can activate PUMA, and caspases-3, -8, and -9 [27,28]. These genes are responsible for the apoptotic mechanism of cell death [29]. Meanwhile, in the case of HeGP2, the presence of silver nanoparticles induces several cytomorphological changes on the liver cancer cells in terms of cell shrinkage and oxidative stress, resulting in apoptosis [30,31]. Moreover, the effect of silver nanoparticles on the inhibition of MCF-7 cells was also reported by Vivek [32] and Jang [33], and again, the apoptosis cell death was reported as a major cause of inhibition. Then, the obtained results in the current study are in line with the reported research in which silver nanoparticles inhibit the cancer cells through the apoptotic mechanism. It is important to mention that the MCF-7 cells are adenocarcinoma (growing in glands of organs), while HCT116 and HePG2 cells are carcinoma; as a result, MCF-7 is a breast cancer model, and its behavior and response to therapies differ from those of other cell types (HCT116 and HePG2).

**Figure 10:**
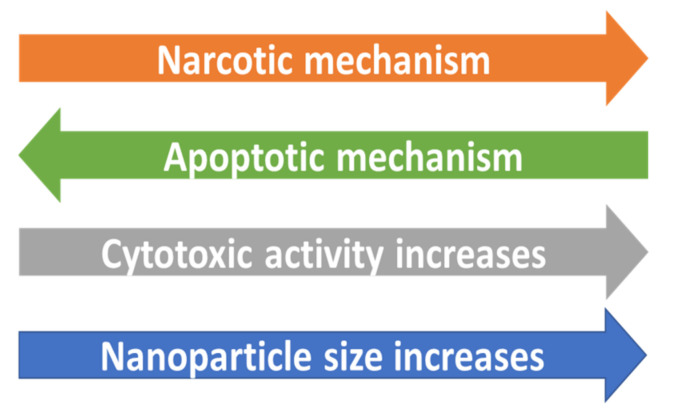
The proposed relationship between the size of the silver nanoparticles and cytotoxic activity and the mechanism of inhibition of the different applied cancer cells in the current study.

The obtained results showed the inhibition of three different types of cancer cells by the prepared Ag-b-SiO_2_ samples. From Figure 5, in the case of HCT 116, a clear trend can be observed; the sample Ag-20 that contained the maximum content of embedded silver nanoparticles exhibited the maximum activity against the HCT 1006 cells. On the other hand, the sample Ag-2 showed less activity than Ag-20. This supports the relationship between the amount of silver nanoparticles and the cytotoxic activity. However, the quantification of the apoptotic and necrotic behavior can predict the relationship between the size of the silver nanoparticles and the inhibition mechanism. The results in Figure 8 showed that in the Ag-2 sample, 80% of the HCT 116 cells exhibited apoptotic inhibition, 20% exhibited late apoptotic inhibition, and almost no necrotic mechanism could be detected; by increasing the silver content, the percentage of apoptotic inhibition decreased, late apoptotic inhibition increased, and also the necrotic mechanism increased but, in total, did not exceed 7% of the total cells. This result shows that when the size of nanoparticles did not exceed 10 nm and the majority of the nanoparticles were embedded within the silica framework, a fast apoptotic inhibition mechanism took place. On the other hand, the same trend could be observed in HeGP2 cells and MCF-7 cells with an exception in the Ag-5 sample in which the percentage of apoptotic cells was maximum. The relationship between the nature and size of silver nanoparticles and the mechanism of inhibition is expressed in Figure 10.

## 4. Materials and Methods

Bio-based silica was obtained from rice husk/straw [17]. The obtained sample was washed several times to remove the contaminations. Then, one gram of the extracted silica was added to 50 mL of a stoichiometric amount of silver nitrate (99%, Sigma) solution. The suspension was stirred for 24 h with a stirring rate of 900 rpm under ambient conditions. The suspension was dried at 100 °C for 24 h, and then the dried solid powder was calcined at 400 °C for 180 min with a heating ramp of 5 degrees/minute followed by hydrogenation in a tube furnace at 300 °C for another 180 min. Finally, the obtained solid powder was sealed, stored in glass vials, and coded as Ag-x (where x is the loading of Ag).

X-ray diffraction (XRD) analysis was carried out using a Schimadzu 6000 DX diffractometer (Shimadzu, Japan) with the aid of a graphite monochromator with Cu-K_α_ (λ = 0.1541 nm). The exact amount of silver was analyzed by using inductively coupled plasma-optical emission spectrometry (ICP-OES) through the ICAP 7000 (1 340 910, Qtegra Software, Germany) instrument. The nitrogen adsorption–desorption isotherms were recorded on a QuantaChrome Autosorb-6B at 77 K. Scanning electron microscopy (SEM, Jeol Model 6360 LVSEM, USA) was used to investigate the morphological structure, and the micrographs were obtained after coating the samples by gold to avoid charging effects and to enhance contrast. Moreover, SEM was equipped with energy-dispersive X-ray analysis (EDX, Jeol Model 6360 LVSEM, USA) that was used as qualitative and semi-quantitative elemental analysis. High-Resolution Transmission Electron Microscopy (HR-TEM) was carried out on a Philips CM30UT electron microscope with a field emission gun as the source of electrons, operated at 300 kV. Samples were mounted on a copper-supported carbon polymer grid by placing a few droplets of a suspension of ground sample in ethanol on the grid, followed by drying under ambient conditions. Finally, the chemical state of metals and surface compositions of materials were analyzed by X-ray photoelectron spectroscopy (XPS). All measurements were carried out at room temperature.

The human cancer cell lines were obtained from Dr. Mohamed Tantawy, Medical and Clinical Studies Institute, National Research Center, Egypt, and provided by American Type Culture Collection (ATCC): breast adenocarcinoma (metastatic mammary gland) (MCF-7) (ATCC^®^ HTB22™), hepatocellular carcinoma (HepG-2) (ATCC^®^ HB8065™), and colon adenocarcinoma (HCT 116) (ATCC^®^ CCL247™). In a humidified, 5% (/*v*/*v*) CO_2_ atmosphere at 37°, cells were maintained in RPMI-1640 supplemented with (100 g/mL), penicillin (100 units/mL), and heat-inactivated fetal bovine serum (10% /*v*/*v*) [18,19].

A sulforhodamine B assay was used to assess the prepared samples’ cytotoxicity against (MCF-7, HepG-2, and HCT 116) human tumor cells (SRB). Amounts of 80% confluent growing cells were trypsinized and cultured for 24 h in a 96-well tissue culture plate before being treated with nanosilver. Untreated cells compared to cells were exposed to the six different concentrations of each compound (0.01, 0.1, 1, 10, and 1000 g/mL) (control). The cells were exposed to the concentrations for 72 h before being fixed with TCA (10% *w*/*v*) for 1 h at 4 °C. After multiple washes, cells were stained for 10 min in the dark with a 0.4 percent (*w*/*v*) SRB solution. Glacial acetic acid, 1% (/*v*/*v*), was used to remove any remaining stain. The SRB-stained cells dissolved in Tris-HCl after drying overnight, and the color intensity was determined in a microplate reader at 540 nm. Using SigmaPlot 12.0 software, the relationship between viability percentage and compound concentrations was analyzed to determine the IC50 (drug dose that reduces survival to 50%) [20].

The DNA binding dyes acridine orange (AO) and ethidium bromide (EtBr) were used to detect viable, apoptotic, and necrotic cells morphologically. When AO intercalates into DNA, it causes both nonviable and viable cells to emit green fluorescence. Nonviable cells take up EtBr, and it emits red fluorescence by intercalating into DNA, while viable cells decline it.

The cells were cultured inside a six-well plate on a cover slide. The cells were treated with IC50 concentrations of nanosilver and incubated for 48 h in a CO_2_ incubator at 37 °C and 5% CO_2_. The cells were washed twice with a cold PBS solution. The cells were stained in each well with a mixture of Acridine Orange 100 g/mL and Ethidium Bromide (AO/EB) 100 g/mL in phosphate-buffered saline (PBS) with a concentration of 1×, and then incubated for 5 min at room temperature (RT) before being examined with a fluorescence microscope [21,22].

## 5. Conclusions

Silver nanoparticles were supported on bio-based silica with different loadings by using silver nitrate solution. The characterization data showed that the systems efficiency by using only water as a solvent was >70%. Moreover, in the small loading samples, only small nanoparticles of silver with an average size of 3–10 nm were obtained, while at high loadings of sliver, extra frameworks of bulky silver (average 200–500 nm) were also observed. The prepared samples exhibited high cytotoxic activity against three kinds of cancer cells. A clear trend was observed, which illustrates a positive relationship between the size of silver nanoparticles and the cytotoxic activity, but a negative relationship between the size and the apoptotic mechanism of cell death.

## Figures and Tables

**Table 1:** The exact amount of silver as obtained from ICP-OE and the textural properties of the prepared as obtained from N_2_ sorption measurements.

Sample	Ag Intended Content	Ag Obtained Content	Surface Area m^2^/g	Pore Volume cm^3^/g	Pore Size nm
Ag-0	0	0	580.1	0.425	4.83
Ag-2	2	1.45	620.2	0.414	4.31
Ag-5	5	3.86	611.3	0.398	4.35
Ag-10	10	7.65	629.6	0.365	3.86
Ag-20	20	13.58	637.4	0.325	3.62

**Table 2:** The IC_50_ (µg) of the prepared Ag-b-SiO_2_ samples against different human solid tumor cells.

Sample	IC_50_ (µg) MCF-7	IC_50_ (µg) HePG2	IC_50_ (µg) HCT 116
Ag-2	5.03 ± 0.2	10.9 ± 0.5	10.1 ± 0.9
Ag-5	3.3 ± 0.3	9 ± 0.9	9.3 ± 0.4
Ag-10	3.02 ± 0.3	8.2 ± 0.14	7.8 ± 0.2
Ag-20	2.4 ± 0.2	8.1 ± 0.4	4.8 ± 0.1

## Data Availability

Not applicable.
